# Elemental Impurities in Pediatric Calcium Carbonate Preparations-High Throughput Quantification and Risk Assessment

**DOI:** 10.3389/fchem.2021.682798

**Published:** 2021-05-17

**Authors:** Chaoqiang Xiao, Li Zhu, Xia Zhang, Rumeng Gao, Shuwang He, Zhihua Lv, Changqin Hu

**Affiliations:** ^1^ Key Laboratory of Marine Drugs, Chinese Ministry of Education, School of Medicine and Pharmacy, Ocean University of China, Qingdao, China; ^2^ Key Laboratory for Quality Research and Evaluation of Chemical Drugs, National Institutes for Food and drug Control, Beijing, China; ^3^ Dyne High-Tech Pediatric Pharmaceutical R&D Institute, Beijing, China

**Keywords:** calcium carbonate, pediatric drugs, element impurities, risk assessment, ICP-MS

## Abstract

Calcium carbonate which is extracted from the Earth in combination with other mineral impurities, is largely used in preparations for pediatric supplements. Elemental impurities in drug products pose toxicological concerns without therapeutic benefits. Thus, it is very urgent to assess the safety of chronic exposure to elements that may be present in trace amounts. In the present study, we developed high throughput ICP-MS method for the quantitative determination of 62 elemental impurities in high matric calcium carbonate samples and validated according to USP 233. Calcium carbonate preparations which state clearly used for child (including neonates, infants, toddlers and children) from 9 manufactures and two types of raw materials (light calcium carbonate and ground calcium carbonate) were investigated in terms of the content and variability of 62 elemental impurities. According to the results, ground calcium carbonate was more suitable to be used in pediatric preparations concerning elemental impurities. Parts of elemental impurities in CaCO_3_ preparations which are derived from the raw materials and the preparation process, may cause potential risks for children. These results indicate that it is necessary to establish a modern instrumental analysis method to evaluate and control elemental impurities in CaCO_3_ raw materials and preparations.

## Introduction

For children, toddlers, and infants, calcium in food often does not meet the needs of the body in this stage of rapid growth and development; since insufficient calcium intake will affect growth and development, potentially leading to diseases such as osteomalacia and rickets (Baker et al., 1999; Lee and Jiang, 2008; Pu et al., 2016), calcium supplement preparations are suggested to meet the body’s demand.

Because of its high calcium content and low price, calcium carbonate is currently recognized as the main calcium source with the highest performance-price ratio in China and other countries, and is thus the preferred raw material for calcium supplement preparations. Calcium carbonate is extracted from the Earth in combination with other minerals, and can be divided into light and ground calcium carbonate according to the production method (Wang et al., 2007). Light calcium carbonate is produced by a chemical processing method, and generally uses limestone and shells as raw materials, whereas ground calcium carbonate is produced by crushing natural calcite directly by a mechanical method.

However, the natural sources of calcium carbonate may contain various elemental impurities such as lead, arsenic, and mercury that are introduced from raw materials or during the production of preparations. Children may be particularly sensitive to the toxic effects of these metals because they tend to absorb a relatively higher fraction of an oral dose than adults. These harmful consequences for children include developmental delays, neurocognitive disorders, behavioral disorders, respiratory problems, cancer, and cardiovascular diseases (Olympio et al., 2009; Brandão and Gontijo, 2012; Al Osman et al., 2019; Jurowski et al., 2019).

In particular, pediatric patients show high sensitivity to lead, which causes irreversible damage to the nervous system that can directly affect intelligence, behavior, and normal development (Measuring Lead Exposure in Infants, Children, and Other Sensitive Populations, 1993; Levin et al., 2008). In adult rats, the absorption of radioactive cerium salts from the gastrointestinal tract ranges from 0.05% to less than 0.1% of the administered dose, whereas suckling rats absorb 40–98% of the administered dose, with the youngest rats retaining the largest percentage of the dose (Kutty et al., 1996). Analysis of the geographic distribution of endemic endomyocardial fibrosis in India suggested a link to high cerium soil concentrations (IRIS Toxicological Review of Cerium Oxide and Cerium Compounds (Interagency Science Discussion Draft), 2009). Metals, especially chromium, cobalt, and nickel, are the most common contact allergens in children (Brandão and Gontijo, 2012), among which cobalt has been regarded as possibly carcinogenic to humans (Colognato et al., 2008). Other elements such as magnesium, manganese, nickel, copper, zinc, and selenium have both nutritional and toxic effects to human health depending on dose (Linshaw et al., 1998; Avula et al., 2010; Singh et al., 2011).

As elemental impurities in drug products pose toxicological concerns without a therapeutic benefit, their levels should be controlled within acceptable limits (Q3D Elemental impurities-guidance for industry, 2020). January 1, 2008, The Guideline for Elemental Impurities (Q3D) of the ICH represents a new paradigm in the control of elemental impurities in pharmaceuticals. The ICH Q3D covers 24 elements, providing permitted daily exposure (PDE) information, which further changed the approach toward control of elemental impurities from the traditional “heavy metals test” to a scientific-based risk assessment using modern analytical instrumentation (Guideline for elemental impurities, 2019). The ICH Q3D is currently the most important guiding principle in the study of elemental impurities, which involves risk assessment for control, and suggests that the inherent characteristics of raw and auxiliary materials from natural sources should be considered in this assessment. The acceptable limits of elemental impurities in drugs as specified by the ICH Q3D are generally set according to the PDE calculated from the no-observed-adverse-effect level or lowest-observed-adverse-effect level in the most relevant animal studies. However, children are not simply a miniature version of adults but rather have different physiological characteristics. Therefore, the limit requirements of the ICH Q3D for elemental impurities in therapeutic drugs may not be completely applicable to pediatric drugs. Thus, the National Food Safety Standard Infant Formula and other guidelines (National Health Commission of the People's Republic of China, 2010; Standard for Infant Formula and Formulations for Special Medical Purposes Intended for Infants, 2007) should be used as a supplement to assess the risk of some elemental impurities for drugs that are commonly taken by young children to best ensure the safety of pediatric medication.

Toward this goal, the aims of this study were to compare the elemental impurity content and variability characteristics of light and ground calcium carbonate to provide guidance for the appropriate selection of calcium carbonate raw materials and to assess the risk of chronic exposure to elements that may be present in trace amounts in supplement preparations for children.

At present, the United States Pharmacopeia (USP), European Pharmacopeia (EP), and Pharmacopeia of the People’s Republic of China (ChP) control elemental impurities of calcium carbonate raw materials mainly through inspection of barium, iron, mercury, and heavy metals (colorimetry), however, the limit of other potential elemental impurities has not been reported. As we known, the colorimetry is not adequate for the purpose of controlling low levels of potentially elemental impurities in drug, and consequently needs to be replaced by highly sensitive instrumental methods such as atomic absorption spectrometry (AAS) (Sheth and Patel, 2020), X-ray fluorescence spectrometry (XRF) (Chowdhury et al., 2020; Sauer et al., 2020), instrumental neutron activation analysis (INAA) (Kamath et al., 2014), inductively coupled plasma-optical emission spectrometry (ICP-OES) (Merusomayajula et al., 2021) and ICP-MS (Chawla et al., 2020). AAS techniques are often restricted by poorer sensitivity and not being multi-element analytical techniques. There are several studies on the application of XRF techniques in pharmaceutical industry, because of the higher detection limits, they are not very popular for quantitative determinations of metal impurities in pharmaceutical samples. INAA is time-consuming, not independent, requires a reactor nearby and involves longer cooling times for detecting certain elements. ICP-OES and ICP-MS, which was recommended in the USP <233> chapter, can be used for simultaneous determination of several elements. However, both of the two methods suffer from matrix and spectral interference. By contrast, the ICP-MS method has advantages of high sensitivity and specificity (Barin et al., 2016; Janchevska et al., 2020).

Therefore, we developed high throughput ICP-MS method for the quantitative determination of 62 elemental impurities in high matric calcium carbonate samples and validated according to USP 233. The limit of each element here was determined by referring to the daily environmental intake levels (Snyder, 1974), PDE values of related elements in the ICH Q3D and the content range in the products. This method was used to evaluate calcium carbonate preparations which state clearly used for child (including neonates, infants, toddlers, children, and adolescents) and two types of raw materials (light and ground calcium carbonate), providing the first comprehensive risk assessment of elemental impurities with limit requirements.

## Material and Methods

### Sample

Two types of calcium carbonate Active Pharmaceutical Ingredient were provided by seven manufacturers. Calcium carbonate preparations, which state clearly used for children (including neonates, infants, toddlers and children), including compound calcium carbonate (effervescent) granules, calcium carbonate D3 (tablets/granules), calcium carbonate, and calcium and zinc gluconate (oral liquid), were available from the Chinese market.

All calcium carbonate preparations have already been tested and they all meet the requirements of the quality standards of ChP 2020.

### Sample Preparation

#### Calcium Carbonate Samples

Two sample processing methods were adopted. In the first method, samples were prepared by dissolving approximately 100 mg of calcium carbonate preparations and calcium carbonate API in a 25 ml volumetric flask with 1.25 ml of concentrated nitric acid and shaking several times to accelerate the dissolution, and then reconstituting with ultrapure water (Milli-Q water purification system) after the reaction is performed about 1 h in room temperature (about 25°C). In the second method, samples were prepared by dissolving approximately 100 mg of calcium carbonate preparations and calcium carbonate API in a 25 ml volumetric flask with 1 ml of concentrated nitric acid and 0.25 ml of hydrochloric acid (CNW Technologies GmbH) with shaking several times to accelerate the dissolution, and then reconstituting with ultrapure water containing 0.1% hydrofluoric acid (HF) after the reaction is performed about 1 h in room temperature (about 25°C). Liquid preparations were tested as a whole vial (5 ml). The test solution was properly diluted as necessary before sample loading. In addition, a blank sample solution was prepared in parallel.

#### Internal Standard

A 500 ng/ml mixture of iridium and ruthenium (Guobiao (Beijing) Testing & Certification Co., Ltd.) was used as the internal standard, which was introduced online into the spray chamber using a peristaltic pump.

#### Calibration Standards

The elemental impurities were divided into two groups. The first group included 42 elements (Li, B, Na, Sc, Ti, V, Fe, Co, Cu, Zn, Ge, As, Se, Br, Rb, Zr, Mo, Ag, Cd, In, Te, Ba, Pr, Ce, Nd, Sm, Tb, Dy, Er, Tm, Yb, Lu, Pb, Bi, Th, U, Mg, Al, Mn, Sr, La, and Os) with 5% nitric acid used as the solvent to prepare a multi-element standard stock solution (S1). The second group included 20 elements (Be, Cr, Ni, Ga, Y, Nb, Rh, Pd, Sn, Sb, Cs, Gd, Ho, Hf, Ta, W, Pt, Au, Hg, Tl) with an acid mixture (HNO_3_:HCl:H_2_O:HF = 4:1:95:0.1) used as the solvent to prepare a multi-element standard stock solution (S2). The single-element standard solutions were obtained from Guobiao (Beijing) Testing & Certification Co., Ltd. except for Os, which was obtained from Aladdin.

The S1 and S2 stock solutions were prepared as a series of standard solutions of 0, 0.5, 1.0, 1.5, and 10 J, respectively, in which J is the limit for each elemental impurity in the final analysis solution.

#### Spiked Samples

Spiked samples were prepared at 50% (0.5J), 100% (1.0J), and 150% (1.5J) of the target limit by spiking 100 mg of the calcium carbonate preparations with 250, 500, and 750 μL of the spiking solution, and diluent filled to a total volume of 25 ml. Six preparations at the 100% spiking level were prepared, and three preparations at the 50 and 150% spiking levels were respectively prepared.

### Instrumentation

All element determinations were performed on an Agilent 7900 ICP-MS system equipped with standard nickel sampling and skimmer cones, a glass concentric nebulizer, quartz spray chamber, and quartz torch with a 2.5 mm internal diameter injector. The instrument also features a collision/reaction cell including a standard helium (He)-mode cell gas line, which provides effective removal of most common polyatomic interferents. The He gas used in collision cells was of high purity (99.999%). Argon gas was used for plasma and dilution at the recommended purity (99.999%). The instrumental experimental parameters for ICP-MS are listed in Table 1. An Agilent SPS-4 Auto-sampler was used to deliver the samples.

**TABLE 1:** Instrument parameters of ICP-MS devices.

Parameter	Setting	Parameter	Setting
Plasma power	1550 W	Sample lifting rate	0.1 rps
Sampling depth	10.0 mm	Spray chamber temperature	2°C
Nebuliser	MicroMist nebulizer	Acquisition mode	Mass spectrum
He gas flow	4.3 ml/min	Peak type	3 points
Carrier gas flow	1.01 L/min	Number of replicates	3

Li, Be, and B were measured using No Gas mode; He gas mode was used for other elements, and the stable time was set to 10 s. The integration time was 0.3 s. Ru (mass number 101) was selected as the internal standard for elements with a mass number below 150, and Ir (mass number 193) was used as the internal standard for elements with a mass number above 150. Table 2 lists the measured mass number of each element. The instruments were tuned systematically. The resolution/mass axis and sensitivity met the measurement requirements of the instruments. Instrument tuning and P/A factor tuning were conducted before batch operation to achieve the best experimental conditions.

**TABLE 2:** Accuracy (mean spike recovery%), repeatability, and intermediate precision (as RSD%).

Element	Isotope	Mode	LOD	LOQ	Recovery (%, *n* = 3)			Repeatability (RSD%, *n* = 6)	Intermediate precision (RSD%, *n* = 12)	J (ppb)
					0.5J	1.0J	1.5J			
Cd	111	He	0.0009	0.003	86.8	101.2	98.8	9.7	9.6	2
Pb	208	He	0.006	0.02	78.4	86.4	88.4	11.0	10.1	2
As	75	He	0.005	0.02	86.4	99.9	99.0	9.9	9.5	4
Hg	201	He	0.02	0.05	100.5	102.0	110.5	3.1	2.9	8
Co.	59	He	0.002	0.008	89.3	100.0	98.3	8.0	8.4	4
V	51	He	0.005	0.02	89.3	99.6	96.1	8.7	8.7	20
Ni	60	He	0.006	0.02	90.3	100.0	103.0	4.3	3.7	4
Tl	205	He	0.004	0.01	87.8	89.8	96.6	7.2	5.4	2
Au	197	He	0.2	0.5	104.4	103.0	106.6	4.3	4.0	20
Pd	105	He	0.003	0.01	93.6	96.1	102.1	7.1	5.1	5
Os	189	He	0.04	0.1	104.4	113.3	135.3	7.8	8.3	0.5
Rh	103	He	0.001	0.005	96.1	97.2	104.2	6.9	5.1	0.4
Se	82	He	0.4	1.3	96.3	107.3	112.4	8.9	8.2	40
Ag	107	He	0.002	0.006	87.4	96.8	95.7	8.5	8.1	2
Pt	195	He	0.002	0.005	93.1	94.0	103.5	7.0	5.7	0.8
Li	7	No gas	0.03	0.1	99.2	97.0	98.7	1.4	17.9	5.0
Sb	121	He	0.002	0.006	93.7	103.9	115.6	7.4	6.1	5
Ba	137	He	0.01	0.03	70.5	88.7	90.2	13.3	12.6	10
Mo	95	He	0.004	0.01	93.1	103.1	101.3	8.0	7.9	8
Cu	63	He	0.03	0.08	89.6	100.2	97.4	8.4	8.4	100
Sn	118	He	0.01	0.03	105.2	106.0	115.4	8.1	5.8	0.4
Cr	52	He	0.01	0.04	99.5	108.3	114.8	4.4	3.5	60
B	11	No gas	0.9	3	88.1	86.1	86.3	1.5	16.4	200
Na	23	He	6	21	72.1	86.8	92.6	13.5	13.2	200
Sc	45	He	0.02	0.1	85.1	94.2	92.1	8.4	8.3	50
Ti	47	He	0.06	0.2	77.6	91.8	90.6	10.0	10.2	50
Fe	56	He	0.6	2	75.8	90.1	88.1	10.1	10.5	800
Zn	66	He	1	4	86.8	95.6	93.1	8.4	8.0	200
Ge	72	He	0.008	0.03	81.1	97.3	95.7	8.6	9.0	4
Br	79	He	1.5	4.9	67.7	81.4	81.7	10.0	9.0	120
Rb	85	He	0.002	0.005	75.4	90.6	87.2	10.6	8.8	0.4
Zr	90	He	0.005	0.02	89.0	98.7	98.1	9.1	8.6	20
In	115	He	0.004	0.01	85.5	95.7	94.9	8.6	8.6	50
Te	125	He	0.01	0.04	83.7	94.7	97.4	10.5	10.6	4
Pr	141	He	0.0003	0.0008	78.2	91.9	93.2	11.0	11.5	0.4
Ce	142	He	0.002	0.008	91.9	103.5	100.7	8.3	8.8	100
Nd	146	He	0.001	0.005	90.2	100.3	97.2	8.6	8.4	20
Sm	147	He	0.0005	0.002	92.6	101.1	100.1	8.5	8.9	8
Tb	159	He	0.0004	0.001	84.8	91.5	91.5	7.8	7.2	2
Dy	163	He	0.0002	0.0008	84.6	92.0	91.8	7.9	7.2	5
Er	166	He	0.0003	0.001	87.7	94.4	93.6	7.8	7.0	8
Tm	169	He	0.00004	0.0001	87.1	94.6	93.6	7.7	6.9	2
Yb	172	He	0.0004	0.001	87.9	95.0	93.8	7.6	6.9	20
Lu	175	He	0.0004	0.001	83.5	90.7	91.1	7.6	6.8	16
Bi	209	He	0.02	0.06	96.8	99.4	91.2	7.0	7.6	0.4
Th	232	He	0.0004	0.001	80.0	91.8	92.9	10.8	10.6	0.24
U	238	He	0.0002	0.0006	68.2	82.0	85.9	20.3	19.9	0.2
Be	9	No gas	0.001	0.004	93.8	102.7	89.6	7.7	14.2	2
Ga	71	He	0.002	0.006	100.7	106.6	114.1	6.9	5.5	4
Y	89	He	0.001	0.004	103.8	107.7	116.9	7.3	5.3	20
Nb	93	He	0.0005	0.002	119.2	119.2	123.5	5.8	6.6	0.4
Cs	133	He	0.0005	0.002	99.9	104.0	113.4	6.7	4.8	0.8
Gd	160	He	0.0006	0.002	103.0	108.2	116.4	3.4	2.8	8
Ho	165	He	0.00008	0.0003	101.4	103.6	112.8	6.4	4.5	0.8
Hf	178	He	0.0009	0.003	110.0	106.9	112.6	6.8	5.1	0.4
Ta	181	He	0.0006	0.002	111.4	107.4	114.2	6.9	5.3	0.2
W	182	He	0.001	0.004	105.3	103.9	112.9	6.8	4.8	2
Mg	24	He	0.6	2	87.7	112.6	98.8	8.4	5.9	1,000
Al	27	He	2	7	90.5	109.0	98.2	6.7	4.7	200
Mn	55	He	0.03	0.1	95.7	108.9	100.6	6.4	4.7	30
Sr	88	He	0.03	0.1	92.3	111.3	100.9	7.1	5.2	100
La	139	He	0.8	3	87.7	102.5	94.2	8.6	7.2	50

The J value was determined according to both the permitted daily exposure (PDA) value of the drug and the dilution multiple during sample preparation. Sample preparation: 100 mg to 25 ml, dilution multiple 4,000 (*n*), J = PDE/*n*.

## Results and Discussion

### Development and Validation of the ICP-MS Method

To achieve a comprehensive assessment of potential elemental impurities in calcium carbonate, there are 62 kinds of target elemental impurities in the study, including most of the elements other than synthetic, radioactive, inert gases and major components of air. The majority of elemental impurity content is in ppb-ppt level. However, ICP-OES is not sensitive enough. The addition of the sample may lead to higher matrix effect and there are too many kinds of impurity and serious line interference. Therefore, ICP-MS method with higher sensitivity is selected in this study. To avoid interactions between elemental impurities, elemental impurities had been properly grouped. A high-throughput method was established for the analysis of 62 elemental impurities in calcium carbonate preparations using different digestion methods. The instrument was equipped with a collision reaction cell. Multiatomic mass spectral interference was eliminated by collision mode of He. As the mass number of Li, Be and B elements is close to that of He, the collision between He and collision reaction cell may lead to undetectable or extremely low response. Therefore, to gain higher sensitivity, No Gas mode was chosen. The dilution of the sample should be considered in combination with the LOD and LOQ of each elemental impurity and matrix interference. Insufficient dilution could cause strong matrix interference. At the same time, it could cause rapid changes in the instrument state. For example, the taper hole accumulation of Ca may lead to the high matrix of the Ca element and poor recovery of elements. On the other hand, over dilution may make higher demands on the LOD of the element. Strongly memory effect of Nb, Ta, and Hf cause LOQ close to their proposed J value under 5% nitric acid matrix. So it’s difficult to meet the detection requirements. Therefore, 0.1% hydrofluoric acid was used to reduce or eliminate the memory effect. With these optimizations, Nb, Ta, and Hf were ultimately detected with excellent limits of quantification (<4 ppb) in He mode. High temperature digestion could cause low recovery of Os and other elements. Hg and other elements need appropriate amount of HCl as a stabilizer (Barin et al., 2016). HCl contains a large amount of Br element, and Ag element reacts with Cl ion to affect the quantity of Ag. This study selected Room temperature digestion. Compared the effects of nitric acid and a mixture of 80:20 nitric acid-hydrochloric acid, simple dissolution in 5% HNO_3_ and HNO_3_/HCl、2% HNO_3_ and HNO_3_/HCl which were selected as appropriate for the detection of different elemental impurities. Results indicated simple dissolution needs more time for the reaction. However, the sample contained a large amount of Ca. To further reduce the influence of matrix effect on the results, the standard curve method of internal standard correction was selected. According to the results of the pre-experiment, Ir and Ru were not detected in the samples. Therefore, these two elements were selected as internal standard in this study.

Except for related elements in the ICH Q3D, the PDE values of other elements was also determined by referring to the daily environmental intake levels and the content range in the products. According to USP<233>, the method validation was based on the J, which was calculated depending on the PDE.

The ICP-MS method was successfully validated in terms of linearity, limit of quantification, accuracy, precision, intermediate precision, specificity, and range to fulfill the requirements for the validation of alternative procedures stated in USP chapter 233. The correlation coefficients for each element were all ≥0.99. The limits of quantitation and range for all elements met the accuracy requirement. The spike recoveries of elements from calcium carbonate preparations are listed in Table 2. Except for the recovery rate of 68% for Br and U at 0.5 J, which was slightly lower than the limit requirement of 70%, all of the other elements met the accuracy requirement of 70–150%. A second analyst prepared and analyzed six preparations of the approximately 100% target limit spiked samples (J) with freshly prepared spiking solutions on a different day. The samples were quantified against fresh calibration standards. Ruggedness was established as operator/day to operator/day precision change in the mean. The results determined for ruggedness are summarized in Table 2. The RSD% for all preparations (*n* = 12) for each target element was ≤25%. All acceptance criteria described in USP chapters 233/232 for ruggedness were met.

### Risk Assessment and Control of Impurity Elements in Calcium Carbonate API Samples

Calcium carbonate is found throughout nature, including in the form of limestone, marble, calcite, and coral. The majority of industrial products containing calcium carbonate are powder materials that are produced through processing the calcium carbonate ore by a mechanical method (i.e., ground calcium carbonate) or a chemical method (i.e., light calcium carbonate). In this study, we assessed light calcium carbonate from four different manufacturers (including seven batches from manufacturer I and one batch each from the other manufacturers) and assessed ground calcium carbonate from three different manufacturers (including four batches from manufacturer V and one batch each from the other manufacturers).

**FIGURE 1:**
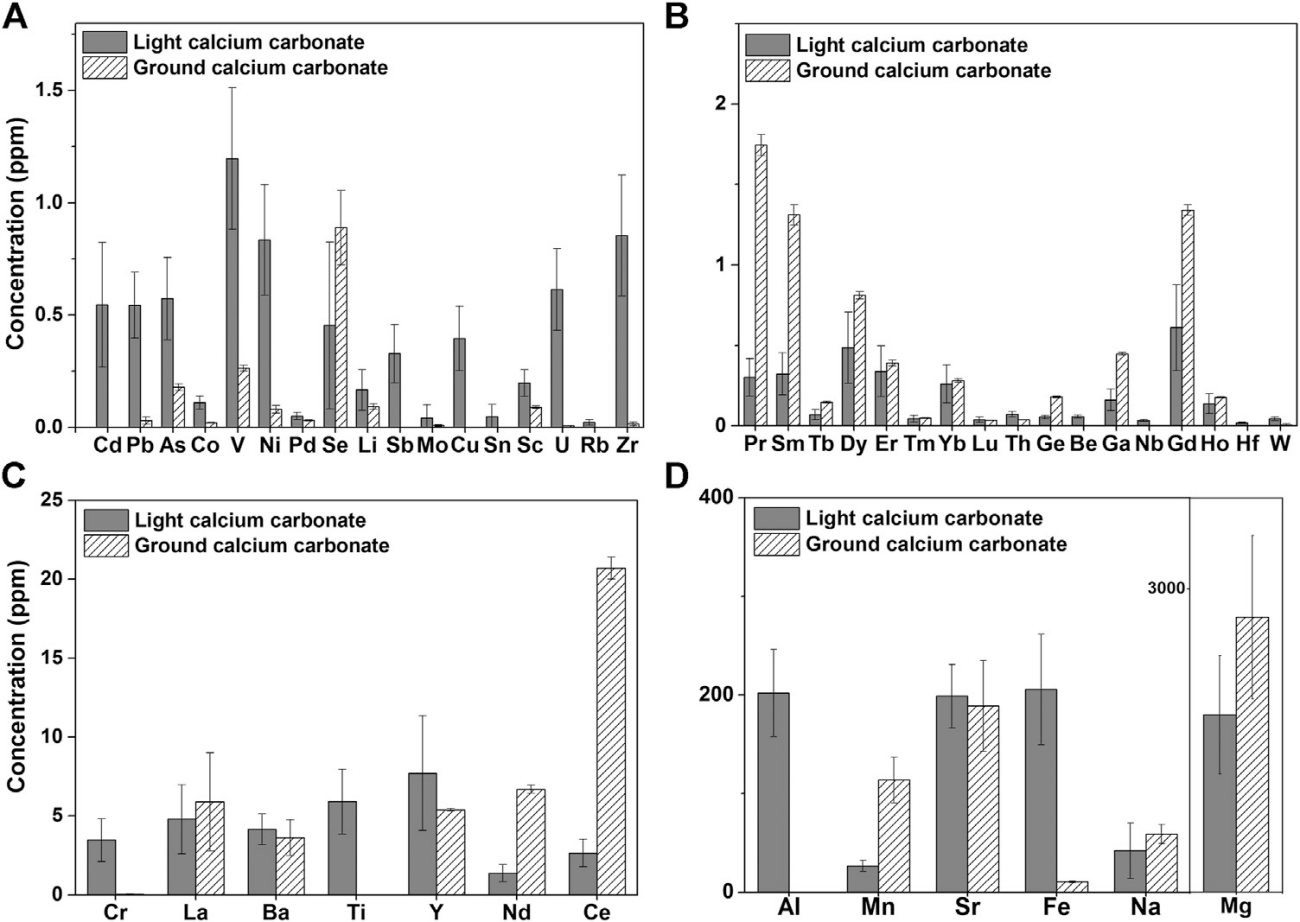
Histogram of Elemental impurity of Light calcium carbonate (manufacturer I, seven batches) and Ground calcium carbonate (manufacturer V, four batches) (Elemental impurities not detected in light calcium and heavy calcium are not listed).

Comparing the test results of light calcium carbonate from manufacturer I and ground calcium carbonate from manufacturer V (Table S1), 15 elemental impurities were not detected in the light calcium carbonate, 23 elemental impurities were not detected in the ground calcium carbonate, and 13 elemental impurities were not detected in either type of calcium carbonate. Taking elemental impurity content within three times the average value as the standard of no difference, we identified 15 elemental impurities with no difference between light and ground calcium carbonate, 22 elemental impurities with higher content in light calcium carbonate than in ground calcium carbonate, and only nine elemental impurities with higher content in ground calcium carbonate than in light calcium carbonate. Specifically, as shown in Figure 1, the contents of Cd, Pb, As, V, Ni, Cr, and Cu in light calcium carbonate were higher than those in ground calcium carbonate, and the values also showed a larger SD in light calcium carbonate (Figure 1A). By contrast, the contents of Ge, Ce, Nd, Sm, Ga, and other elements were higher in ground calcium carbonate than those in light calcium carbonate, but the SD was smaller than that in light calcium carbonate (Figures 1B,C). Among them, the content of Ce in ground calcium carbonate was approximately 10 times higher than that in light calcium carbonate. In addition, the content of Mn in ground calcium carbonate was 114 ± 23 ppm, whereas that in light calcium carbonate was 28 ± 6 ppm, indicating both a higher mean and SD in ground calcium carbonate (Figure 1D). The differences between the two types of calcium carbonate were manifested in the quantity, content, and batch difference of their constituent elemental impurities. This overall comparison of the detection results of light calcium carbonate from manufacturer I and ground calcium carbonate from manufacturer V showed that ground calcium carbonate is slightly superior to light calcium carbonate in terms of the content, quantity, and variation degree of elemental impurities, whereas the content and variation of different elemental impurities had no absolute correlation with calcium carbonate type.

Among the 24 elements specified in the ICH Q3D, 12 impurities in light calcium carbonate from manufacturer I were detected at higher levels than those in ground calcium carbonate from manufacturer V. In particular, the levels of the primary elements Cd (0.6 ± 0.3 ppm) and Pb (0.5 ± 0.1 ppm) were higher than the limit requirements (0.5 ppm, based on a daily dose of 10 g), although the levels of other elements were lower than the control thresholds, with some notable variation. For elements not specified in the Q3D, we referred to the data on environmental exposure (Snyder, 1974) and set the limit far lower than the environmental exposure. Among these elements, La (∼5 ppm), Sr (∼200 ppm), Yb (∼0.3 ppm), Dy (∼0.7 ppm), and Er (∼0.4ppm) were detected in both types of calcium carbonate with comparable content. The contents of Pr, Ce, and Nd in ground calcium carbonate were higher than the proposed limit value, and the contents of PR, Ce, and Nd differed substantially between light and ground calcium carbonate. For such elemental impurities without clearly defined limit requirements, in-depth toxicological studies should be conducted to establish reasonable limits.

At present, the Fe content in calcium carbonate raw materials is mainly controlled by comparison with a standard iron solution through a color reaction. The ChP specifies that the Fe content should not exceed 400 ppm, whereas the EP specifies a limit of 200 ppm. Using our ICP-MS method, the Fe content in light calcium carbonate products from manufacturer IV was 424 ppm, which is close to or higher than the limit requirement, and it was difficult to distinguish accurately by visual colorimetry.

**FIGURE 2:**
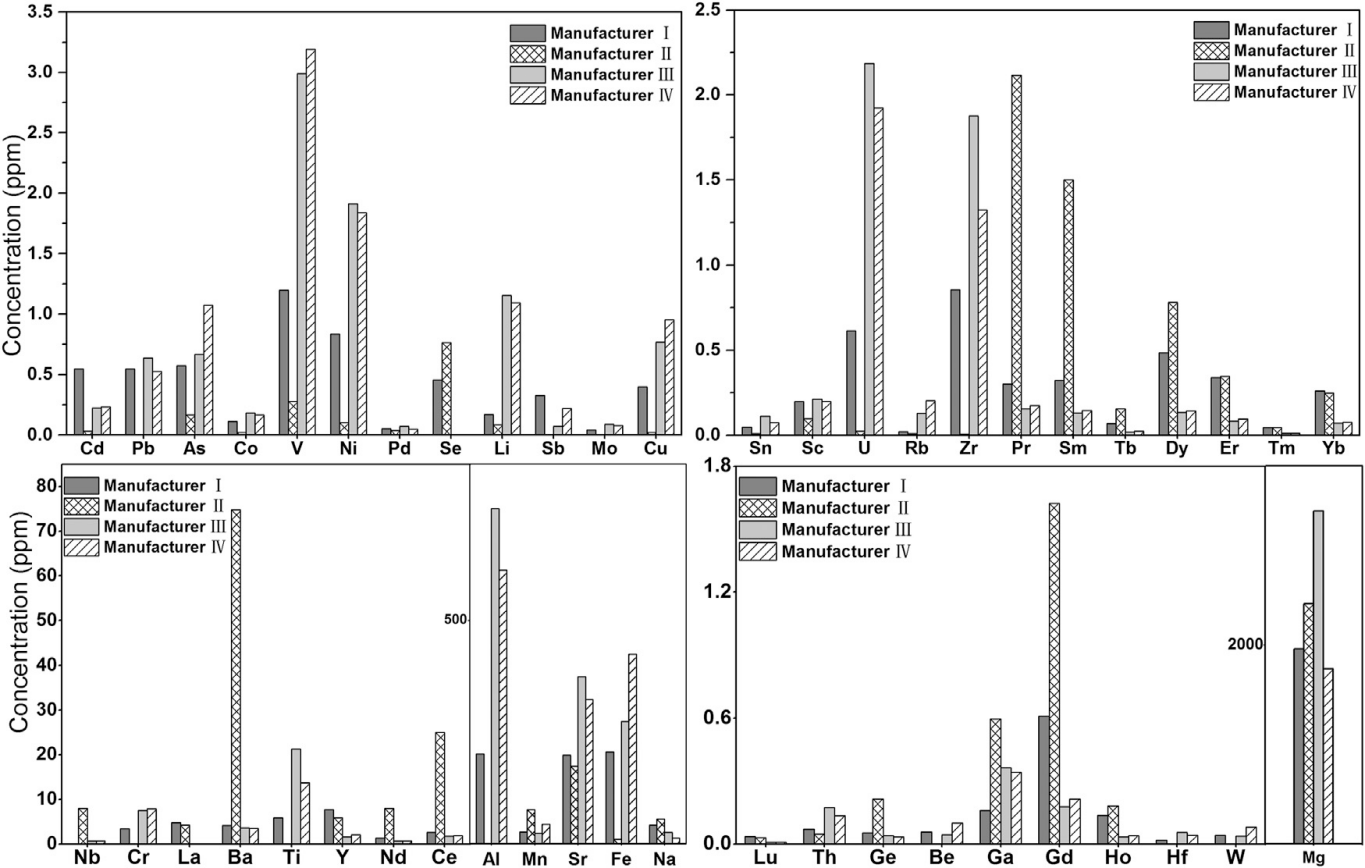
Histogram of Elemental impurity of Light calcium carbonate (manufacturer I ∼ manufacturer IV) (Elemental impurities not detected in light calcium are not listed).

Importantly, the content of each elemental impurity detected in light calcium carbonate products from different manufacturers varied greatly (Figure 2). The Cd content in products from manufacturer I was 0.6 ppm, while that in products from manufacturer II was only 0.03 ppm. Other elemental impurities such as Pb (0–0.6 ppm), As (0.2–1.1 ppm), Fe (10–424 ppm), and La (0–5 ppm) also showed great content variations among products from different manufacturers. Among the ground calcium carbonate products from three manufacturers (Supplementary Table S1), the Ba (2,629 ppm) content of ground calcium carbonate from manufacturer VI did not meet the standard in the pharmacopoeia (ChP and USP), which is consistent with the detection results of this method. The La element in the ground calcium carbonate from manufacturer VIII was 26 ppm higher than that of other manufacturers (about 6 ppm), whereas the content of each elemental impurity in the ground calcium carbonate products from other manufacturers was consistent.

This observed difference in elemental impurities between light and ground calcium carbonate may be due to the fact that light calcium carbonate is generally produced by calcining natural limestone from broad sources, whereas ground calcium carbonate is generally produced by grinding natural calcite with high whiteness and purity. The sources of limestone as the raw material of calcium carbonate preparations may vary among manufacturers, resulting in large variations in the elemental impurities in the light calcium carbonate produced by different manufacturers, whereas the elemental impurities in ground calcium carbonate prepared from calcite are relatively stable. Therefore, from the perspective of quality control, ground calcium carbonate is recommended as the raw material of calcium supplement preparations. In addition, considering the uncertainty about raw material sources, corresponding quality control methods should be established for both light and ground calcium carbonate, and only raw materials that meet the requirements for the production of preparations should be selected so as to ensure high quality.

### Risk Assessment and Control of Impurity Elements in Calcium Carbonate Preparations

The elemental impurities of toxicological concern detected in multiple batches of calcium carbonate preparations from nine manufacturers all met the Q3D limit requirements. However, the limits of some elemental impurities differ from those of mineral indicators in infant formula food standards; therefore, these preparations may have certain risks when given to young children. The detailed test results are summarized in Supplementary Table S2. For example, the Se content in the products from manufacturers P1 (0.5 ± 0.06 ppm) and P9 (0.4 ± 0.03 ppm) exceeded the 0.06 ppm limit specified by infant formula food standards, but was far lower than the 15 ppm limit specified by the Q3D. In all products tested, the content of the heavy metal Cu did not exceed the limit of 0.9 ppm specified by infant formula food standards or the much higher limit requirement of the Q3D (300 ppm). In view of these mineral elements with different specified limits, it is suggested to attempt to meet the nutritional requirements only so as to avoid possible safety risks caused by an excessive dosage, especially for young children and infants.

The preparation from manufacturer P4 was a calcium supplement preparation specifically designed for children, and the Pb content was slightly higher than the control threshold, whereas the Pb content in preparation products from other manufacturers was 30% lower than the limit. The content of Pb in multiple batches of raw materials was above 0.5 ppm with a maximum of 0.2 ppm. These results demonstrated that the main source of Pb in the preparations was the raw materials, whereas the auxiliary materials had only a low or no contribution to the elemental impurities. The content of Pb in preparations from different manufacturers ranged from 0.01 to 0.2 ppm, indicating high variation among different manufacturers. Blood Pb levels above 10 μg/dl are known to affect various areas of the brain that influence behavior and cause many other health problems in children (Flannery et al., 2020). Therefore, an exclusive method should be adopted to ensure that the content of Pb meets the limit requirements. Hg, Os, and Pt were not detected in any of the preparations.

Although the contents of other elements varied among different products, they were all far lower than the Q3D limits. However, it is worth noting that the content of Na was 26,000 ppm in products from manufacturer P3 and was approximately 2,000 ppm in products from manufacturer P4. The content of Na in products from other manufacturers ranged between 25 ± 3 ppm and 258 ± 16 ppm, but was lower than 100 ppm in many batches of raw materials, and did not differ between the light and ground calcium carbonate. However, USP and ChP pharmacopoeia are no relevant inspection item for Na in the standards for calcium carbonate preparations, only alkali metal inspection items. According to the standards of the pharmacopoeia, calcium is precipitated from calcium carbonate raw materials by an ammonium oxalate test solution, sulfuric acid is added to the filtered solution for the reaction, and finally the levels of Mg and alkali metals (Li, Na, K, Rb, and Cs) in calcium carbonate raw materials are controlled based on an ignition residue not exceeding 1.0%. we know that the content of Na for meeting normal nutritional needs specified in the infant formula food standards for infants aged 0–12 months is 120–410 ppm (National Health Commission of the People's Republic of China, 2010; Standard for Infant Formula and Formulations for Special Medical Purposes Intended for Infants, 2007). The pharmacopoeia method obviously cannot meet the current testing requirements. Accordingly, the daily dosage for infants described in the instructions of preparations from manufacturers P3 and P4 are likely to cause Na to exceed the appropriate intake, imposing a great health risk. The variation in Na content among products is likely derived from its introduction in the preparation process. Sodium-containing excipients are commonly used in calcium carbonate preparations to avoid rapid precipitation of calcium carbonate. However, excessive Na causes hypertension and increases the excretion burden of the kidneys, which may lead to water and salt metabolic disorders in infants and toddlers, and may even cause organ injuries (Appel et al., 2015). Therefore, calcium carbonate preparations for children should avoid the use of sodium-containing excipients, and further research is needed to further evaluate Na as an introduced element in preparation products so as to avoid any risks of intake by young children.

The Fe content in calcium carbonate raw materials is controlled through specific inspection items, but no relevant control items are specified for preparation products. The content of Fe in preparations from manufacturer P4 and that in the light calcium carbonate raw material from manufacturer IV was 472 and 424 ppm, respectively, which were close to the limit requirements for raw materials, indicating that Fe might be introduced mainly from the raw materials or preparation processes. The content of Fe in the products from manufacturer P4 was far higher than the limit requirement of 11 ppm in infant formula food (National Health Commission of the People's Republic of China, 2010; Standard for Infant Formula and Formulations for Special Medical Purposes Intended for Infants, 2007), indicating certain risks for infants.

Overall, these results suggest that the elemental impurities in calcium carbonate preparations, such as Na and Fe, are mainly introduced in the preparation process as well as from the raw materials. However, there are no relevant inspection items in current drug standards or corresponding statements in the preparation instructions, which can impose an unknown risk to young children. Therefore, inspection items for Na, Fe, and other elemental impurities should be added for calcium carbonate preparations, especially those labeled specifically as “for use in children.” ICP-MS is a modern analytical method recommended by the USP 233. Therefore, ICP-MS should be selected as an alternative to traditional methods for evaluations and control of the elemental impurities in calcium carbonate from mineral sources and its preparation products.

## Conclusion

The high-throughput ICP-MS method for the quantitative determination of 62 elemental impurities in high matric calcium carbonate samples were developed and used to evaluate calcium carbonate preparations used for child (including neonates, infants, toddlers, children, and adolescents) and two types of raw materials (light and ground calcium carbonate).

Results in raw materials indicated testing should be required for each batch because the levels of these impurities varied significantly from one batch to another due to variations in source. The elemental impurities which cause toxicological concerns in calcium carbonate products intended for children all met the current Q3D limit requirements, marked variations were found between different manufacturers and batches, especially for Pb, Na, and Fe. Specific inspection items should be set up, and modern analysis methods such as ICP-MS should be adopted for accurate quality control so as to ensure the safety of long-term calcium supplementation in children.

Our results indicate the pediatric drugs should adhere to the concept of “tailor-made” and the quality standards for pediatric drugs should be formulated in addition to flavor and dosage forms to ensure the safety of pediatric medication, especially for calcium supplements which are commonly used.

## Data Availability

The original contributions presented in the study are included in the article/Supplementary Material, further inquiries can be directed to the corresponding authors.
