# Supplementary material for: Elemental Impurities in Pediatric Calcium Carbonate Preparations-High Throughput Quantification and Risk Assessment
Source: Front Chem. 2021 May 17;9:682798. doi: 10.3389/fchem.2021.682798 (PMC8173443; doi:10.3389/fchem.2021.682798)
Supplement: Supplementary file 1 [file DataSheet1.docx]

**Elemental Impurities in Pediatric Calcium Carbonate Preparations-high throughput quantification a< LOQ Risk Assessment**

**Supplementary Material**

TableS1 Elemental impurities determined in light calcium carbonate and ground calcium carbonate

| **Elemental** | **Light calcium carbonate** | | | | **Ground calcium carbonate** | | | **Limit (ppm)** |
| --- | --- | --- | --- | --- | --- | --- | --- | --- |
|  | Manufacturer  Ⅰ(n=7) | Manufacturer  Ⅱ | Manufacturer  Ⅲ | Manufacturer  Ⅳ | Manufacturer  Ⅴ(n=4) | Manufacturer  Ⅵ | Manufacturer  Ⅷ |  |
| Cd | 0.6±0.7 | 0.03 | 0.2 | 0.2 | < LOQ | 0.002 | 0.001 | 0.5 |
| Pb | 0.5±0.1 | < LOQ | 0.6 | 0.5 | 0.03±0.02 | 0.02 | 0.03 | 0.5 |
| As | 0.6±0.2 | 0.2 | 0.7 | 1 | 0.2±0.02 | 0.2 | 0.2 | 1.5 |
| Hg | < LOQ | < LOQ | < LOQ | < LOQ | < LOQ | < LOQ | < LOQ | 3 |
| Co | 0.1±0.03 | 0.02 | 0.2 | 0.2 | 0.02±0.001 | 0.02 | 0.02 | 5 |
| V | 1±0.3 | 0.3 | 3 | 3 | 0.3±0.01 | 0.4 | 0.3 | 10 |
| Ni | 0.8±0.2 | 0.1 | 2 | 2 | 0.08±0.01 | 0.1 | 0.2 | 20 |
| Tl | < LOQ | 0.002 | 0.01 | 0.008 | < LOQ | 0.005 | 0.004 | 0.8 |
| Au | < LOQ | < LOQ | < LOQ | < LOQ | < LOQ | < LOQ | < LOQ | 10 |
| Pd | 0.05±0.01 | 0.04 | 0.07 | 0.05 | 0.03±0.002 | 0.06 | 0.04 | 10 |
| Os | < LOQ | < LOQ | < LOQ | < LOQ | < LOQ | < LOQ | < LOQ | 10 |
| Rh | < LOQ | 0.001 | 0.001 | 0.001 | < LOQ | 0.001 | 0.001 | 10 |
| Se | < LOQ | 0.8 | < LOQ | < LOQ | 0.9±0.2 | 0.8 | 0.7 | 15 |
| Ag | 0.01±0.00 | 0.003 | 0.01 | 0.008 | < LOQ | < LOQ | < LOQ | 15 |
| Pt | < LOQ | < LOQ | 0.000 | < LOQ | < LOQ | < LOQ | < LOQ | 10 |
| Li | 0.2±0.08 | 0.08 | 1 | 1 | 0.09±0.01 | 0.4 | 0.2 | 55 |
| Sb | 0.3±0.1 | 0.003 | 0.07 | 0.2 | < LOQ | 0.003 | 0.005 | 120 |
| Ba | 4±1 | 75 | 4 | 4 | 4±1 | 2629 | 9 | 140 |
| Mo | 0.04±0.05 | < LOQ | 0.09 | 0.08 | 0.01±0.004 | 0.01 | 0.006 | 300 |
| Cu | 0.4±0.1 | 0.02 | 0.8 | 1 | < LOQ | 0.05 | 0.07 | 300 |
| Sn | 0.04±0.05 | 0.008 | 0.1 | 0.07 | < LOQ | 0.003 | 0.005 | 600 |
| Cr | 4±1 | 0.07 | 7 | 8 | 0.04±0.01 | 0.1 | 0.2 | 1100 |

Table S1 (continued)

| B | < LOQ | < LOQ | 1 | 0.9 | < LOQ | < LOQ | < LOQ | 50 |
| --- | --- | --- | --- | --- | --- | --- | --- | --- |
| Na | 47±28 | 55 | 25 | 13 | 59±10 | 93 | 69 | 50 |
| Sc | 0.2±0.06 | 0.1 | 0.2 | 0.2 | 0.09±0.01 | 0.2 | 0.2 | 12.5 |
| Ti | 6±2 | < LOQ | 21 | 14 | < LOQ | < LOQ | < LOQ | 12.5 |
| Fe | 207±51 | 10 | 274 | 424 | 11±0.6 | 23 | 25 | 200 |
| Zn | < LOQ | < LOQ | 3 | 3 | < LOQ | < LOQ | < LOQ | 50 |
| Ge | 0.05±0.01 | 0.2 | 0.04 | 0.03 | 0.2±0.01 | 0.2 | 0.2 | 1 |
| Br | < LOQ | 1 | < LOQ | 1 | 1±0.2 | 2 | 1 | 30 |
| Rb | 0.02±0.01 | 0.009 | 0.1 | 0.2 | < LOQ | 0.04 | 0.03 | 0.1 |
| Zr | 0.8±0.2 | 0.006 | 2 | 1 | 0.02±0.01 | 0.01 | 0.03 | 5 |
| In | < LOQ | < LOQ | < LOQ | < LOQ | < LOQ | < LOQ | < LOQ | 12.5 |
| Te | < LOQ | < LOQ | < LOQ | < LOQ | < LOQ | < LOQ | < LOQ | 1 |
| Pr | 0.3±0.1 | 2 | 0.2 | 0.2 | 1.8±0.07 | 2 | 2 | 0.1 |
| Ce | 3±0.80 | 25 | 2 | 2 | 21±0.7 | 23 | 22 | 25 |
| < LOQ | 1.4±0.5 | 8 | 0.7 | 0.7 | 7±0.3 | 8 | 7 | 5 |
| Sm | 0.3±0.1 | 2 | 0.1 | 0.1 | 1±0.06 | 2 | 1 | 2 |
| Tb | 0.07±0.03 | 0.2 | 0.02 | 0.02 | 0.1±0.005 | 0.1 | 0.1 | 0.5 |
| Dy | 0.5±0.2 | 0.8 | 0.1 | 0.1 | 0.8±0.02 | 0.8 | 0.8 | 1.25 |
| Er | 0.4±0.2 | 0.3 | 0.08 | 0.1 | 0.4±0.02 | 0.4 | 0.4 | 2 |
| Tm | 0.05±0.02 | 0.04 | 0.01 | 0.01 | 0.05±0.002 | 0.05 | 0.05 | 0.5 |
| Yb | 0.3±0.1 | 0.2 | 0.07 | 0.07 | 0.3±0.01 | 0.3 | 0.3 | 5 |
| Lu | 0.04±0.02 | 0.03 | 0.009 | 0.01 | 0.03±0.001 | 0.04 | 0.04 | 4 |
| Bi | < LOQ | < LOQ | 0.02 | < LOQ | < LOQ | < LOQ | < LOQ | 0.1 |
| Th | 0.07±0.02 | 0.05 | 0.2 | 0.1 | 0.04±0.001 | 0.1 | 0.1 | 0.06 |
| U | 0.6±0.2 | 0.02 | 2 | 2 | 0.01±0.001 | 0.03 | 0.02 | 0.05 |
| Be | 0.06±0.01 | 0.001 | 0.05 | 0.1 | < LOQ | 0.008 | 0.007 | 0.5 |

Table S1 (continued)

| Ga | 0.2±0.07 | 0.6 | 0.4 | 0.3 | 0.4±0.01 | 0.4 | 0.5 | 1 |
| --- | --- | --- | --- | --- | --- | --- | --- | --- |
| Y | 8±3 | 6 | 2 | 2 | 5.39±0.09 | 4.679 | 5.729 | 5 |
| Nb | 0.03±0.01 | 0.0005 | 0.1 | 0.1 | < LOQ | 0.0002 | 0.001 | 0.1 |
| Cs | 0.005±0.002 | 0.006 | 0.04 | 0.05 | 0.003±0.001 | 0.02 | 0.01 | 0.2 |
| Gd | 0.6±0.2 | 2 | 0.2 | 0.2 | 1±0.03 | 1 | 1 | 2 |
| Ho | 0.1±0.06 | 0.2 | 0.04 | 0.04 | 0.2±0.003 | 0.2 | 0.2 | 0.2 |
| Hf | 0.02±0.004 | 0.002 | 0.06 | 0.04 | < LOQ | 0.002 | 0.002 | 0.1 |
| Ta | 0.002±0.0001 | 0.0002 | 0.006 | 0.005 | < LOQ | 0.0002 | 0.0005 | 0.05 |
| W | 0.04±0.01 | 0.002 | 0.04 | 0.08 | 0.01±0.01 | 0.002 | 0.004 | 0.5 |
| Mg | 2,137±644 | 2416 | 3347 | 1762 | 2,765±674 | 1867 | 1870 | 250 |
| Al | 194±45 | < LOQ | 750 | 613 | < LOQ | <LOQ | < LOQ | 50 |
| Mn | 28±6 | 77 | 24 | 44 | 114±23 | 67 | 62 | 7.5 |
| Sr | 205±33 | 174 | 374 | 324 | 189±46 | 248 | 195 | 25 |
| La | 5±3 | 4 | < LOQ | < LOQ | 6±3 | 7 | 26 | 12.5 |

Note: “< LOQ”: below the respective LOQs.

Table S2 Elemental impurities determined in calcium carbonate preparations

| **ICH Q3D classification** | | | **Element** | | **Manufacturer** | | | | | | | | | | | | | | | | |  |  |  |
| --- | --- | --- | --- | --- | --- | --- | --- | --- | --- | --- | --- | --- | --- | --- | --- | --- | --- | --- | --- | --- | --- | --- | --- | --- |
|  |  |  |  |  | P1 | | P2 | | P3 | | P4 | | P5 | | P6 | | P7 | | P8 | | P9 |  |  |  |
|  |  |  |  |  |  |  |  |  |  |  |  |  |  |  |  |  |  |  |  |  |  |  | | |
| 1 | | | Cd | | 0.0008±0.0001 | | 0.06±0.09 | | < LOQ | | 0.007±0.0004 | | 0.002±0.0007 | | 0.0002±0.0001 | | < LOQ | | 0.1±0.01 | | 0.003±0.002 |  | | |
| 1 | | | Pb | | 0.008±0.003 | | 0.2±0.04 | | < LOQ | | 0.2±0.02 | | 0.01±0.002 | | 0.01±0.001 | | 0.01±0.003 | | 0.07±0.004 | | 0.02±0.0003 |  | | |
| 1 | | | As | | 0.09±0.01 | | 0.2±0.03 | | 0.05±0.01 | | 0.3±0.04 | | 0.02±0.003 | | 0.002±0.00002 | | 0.06±0.01 | | 0.06±0.01 | | 0.07±0.01 |  | | |
| 1 | | | Hg | | < LOQ | | < LOQ | | < LOQ | | < LOQ | | < LOQ | | < LOQ | | < LOQ | | < LOQ | | < LOQ |  | | |
| 2A | | | Co | | 0.01±0.0002 | | 0.03±0.01 | | 0.006±0.003 | | 0.07±0.01 | | < LOQ | | 0.0002±0.0001 | | 0.01±0.003 | | 0.02±0.002 | | 0.02±0.001 |  | | |
| 2A | | | V | | 0.1±0.01 | | 0.4±0.09 | | 0.1±0.01 | | 0.3±0.03 | | 0.07±0.005 | | 0.006±0.0004 | | 0.1±0.01 | | 0.2±0.01 | | 0.1±0.02 |  | | |
| 2A | | | Ni | | 0.03±0.0006 | | 0.2±0.07 | | 0.05±0.02 | | 0.2±0.01 | | < LOQ | | 0.002±0.0001 | | 0.08±0.02 | | 0.2±0.01 | | 0.1±0.004 |  | | |
| 2B | | | Tl | | 0.01±0.002 | | 0.006±0.0009 | | 0.004±0.0006 | | 0.003±0.0003 | | 0.002±0.0004 | | <LOQ | | 0.0043±0.0011 | | 0.0040±0.0005 | | 0.0040±0.0002 |  | | |
| 2B | | | Au | | 0.2±0.04 | | 0.09±0.01 | | 0.06±0.01 | | < LOQ | | < LOQ | | < LOQ | | 0.05±0.02 | | 0.06±0.01 | | < LOQ |  | | |
| 2B | | | Pd | | 0.01±0.002 | | 0.008±0.001 | | 0.005±0.002 | | 0.02±0.0007 | | 0.005±0.0007 | | < LOQ | | 0.02±0.002 | | 0.02±0.004 | | 0.04±0.01 |  | | |
| 2B | | | Os | | < LOQ | | < LOQ | | < LOQ | | < LOQ | | < LOQ | | < LOQ | | < LOQ | | < LOQ | | < LOQ |  | | |
| 2B | | | Rh | | < LOQ | | < LOQ | | < LOQ | | < LOQ | | < LOQ | | < LOQ | | < LOQ | | < LOQ | | < LOQ |  | | |
| 2B | | | Se | | 0.5±0.06 | | < LOQ | | < LOQ | | < LOQ | | < LOQ | | < LOQ | | < LOQ | | < LOQ | | 0.4±0.03 |  | | |
| 2B | | | Ag | | < LOQ | | < LOQ | | < LOQ | | < LOQ | | < LOQ | | < LOQ | | < LOQ | | 0.002±0.0005 | | < LOQ |  | | |
| 2B | | | Pt | | < LOQ | | < LOQ | | < LOQ | | < LOQ | | < LOQ | | < LOQ | | < LOQ | | < LOQ | | < LOQ |  | | |
| 2B | | | Li | | 0.08±0.004 | | 0.2±0.07 | | 0.07±0.01 | | 0.1±0.001 | | 0.2±0.01 | | 0.003±0.0001 | | < LOQ | | 0.04±0.004 | | 0.2±0.01 |  | | |
| 2B | | | Sb | | 0.006±0.001 | | 0.06±0.04 | | 0.005±0.002 | | 0.008±0.0008 | | 0.005±0.0005 | | 0.0004±0.0001 | | 0.004±0.0008 | | 0.004±0.0005 | | 0.005±0.0002 |  | | |
| 3 | | | Ba | | 28±23 | | 1±0.1 | | 0.8±0.3 | | 1±0.1 | | 0.07±0.01 | | 0.01±0.0009 | | 3±0.6 | | 1±0.7 | | 10±0.6 |  | | |
| 3 | | | Mo | | 0.02±0.01 | | 0.02±0.01 | | 0.01±0.002 | | 0.2±0.03 | | 0.01±0.002 | | < LOQ | | 0.01±0.004 | | 0.006±0.003 | | 0.03±0.01 |  | | |
| 3 | | | Cu | | 0.06±0.01 | | 0.15±0.03 | | 0.03±0.01 | | 0.3±0.03 | | 0.07±0.01 | | 0.01±0.01 | | 0.21±0.05 | | 0.1±0.02 | | 0.4±0.2 |  | | |
| 3 | | | Sn | | 0.006±0.003 | | 0.02±0.002 | | 0.003±0.0002 | | 0.009±0.001 | | 0.008±0.002 | | 0.3±0.003 | | 0.05±0.05 | | 0.03±0.01 | | 0.05±0.01 |  | | |
| 3 | Cr | | 0.07±0.01 | | 0.8±0.4 | | 0.08±0.02 | | 0.1±0.01 | | 0.04±0.001 | | 0.007±0.0002 | | 0.1±0.02 | | 0.4±0.03 | | | 0.2±0.05 | |  | |  |
| - | B | | < LOQ | | < LOQ | | < LOQ | | 0.8±0.02 | | < LOQ | | < LOQ | | < LOQ | | < LOQ | | | < LOQ | |  | |  |
| - | Na | | 58±3 | | 25±3 | | 25,970±8,963 | | 1,911±137 | | 258±16 | | 168±2 | | 40±5 | | 39±2 | | | 78±5 | |  | |  |
| - | Sc | | 0.08±0.01 | | 0.05±0.01 | | < LOQ | | 0.2±0.03 | | < LOQ | | < LOQ | | 0.05±0.01 | | 0.04±0.001 | | | 0.08±0.01 | |  | |  |
| - | Ti | | 0.3±0.03 | | 3±0.9 | | < LOQ | | 2±0.3 | | < LOQ | | 0.003±0.0004 | | 0.4±0.09 | | 0.6±0.2 | | | 0.1±0.04 | |  | |  |
| - | Fe | | 14±1 | | 66±12 | | 5±2 | | 472±56 | | < LOQ | | < LOQ | | 17±3 | | 52±1 | | | 19±1 | |  | |  |
| - | Zn | | < LOQ | | < LOQ | | < LOQ | | < LOQ | | < LOQ | | 366±4 | | < LOQ | | 1±0.1 | | | 2±1 | |  | |  |
| - | Ge | | 0.1±0.01 | | 0.02±0.01 | | 0.05±0.01 | | 0.03±0.004 | | < LOQ | | < LOQ | | 0.1±0.01 | | 0.02±0.002 | | | 0.1±0.00002 | |  | |  |
| - | Br | | 5±2 | | 2±0.4 | | < LOQ | | < LOQ | | 2±0.04 | | 0.06±0.01 | | < LOQ | | < LOQ | | | < LOQ | |  | |  |
| - | Rb | | 0.02±0.0009 | | 0.02±0.004 | | 0.01±0.0008 | | 0.04±0.01 | | 0.1±0.01 | | 0.007±0.0003 | | 0.01±0.002 | | 0.009±0.002 | | | 0.05±0.01 | |  | |  |
| - | Zr | | 0.2±0.01 | | 0.2±0.05 | | < LOQ | | 0.8±0.2 | | < LOQ | | 0.0004±0.0001 | | 0.1±0.02 | | 0.03±0.003 | | | 0.1±0.2 | |  | |  |
| - | In | | < LOQ | | < LOQ | | < LOQ | | < LOQ | | < LOQ | | 0.001±0.0001 | | < LOQ | | < LOQ | | | < LOQ | |  | |  |
| - | Te | | < LOQ | | < LOQ | | < LOQ | | < LOQ | | < LOQ | | < LOQ | | < LOQ | | < LOQ | | | < LOQ | |  | |  |
| - | Pr | | 1±0.05 | | 0.05±0.04 | | 0.4±0.1 | | 0.1±0.01 | | < LOQ | | 0.001±0.00001 | | 1±0.1 | | 0.1±0.01 | | | 1±0.06 | |  | |  |
| - | Ce | | 11±0.6 | | 0.6±0.3 | | 5±2 | | 2±0.2 | | < LOQ | | 0.01±0.0002 | | 13±2 | | 1±0.05 | | | 14±0.8 | |  | |  |
| - | < LOQ | | 4±0.2 | | 0.2±0.2 | | 2±0.5 | | 0.6±0.06 | | < LOQ | | 0.006±0.0002 | | 4±0.5 | | 0.6±0.03 | | | 4±0.2 | |  | |  |
| - | Sm | | 0.7±0.04 | | 0.05±0.04 | | 0.3±0.1 | | 0.1±0.02 | | < LOQ | | 0.002±0.0001 | | 0.8±0.1 | | 0.1±0.005 | | | 0.9±0.03 | |  | |  |
| - | Tb | | 0.07±0.004 | | 0.01±0.01 | | 0.03±0.01 | | 0.02±0.002 | | < LOQ | | 0.0003±0.00003 | | 0.08±0.01 | | 0.02±0.001 | | | 0.08±0.003 | |  | |  |
| - | Dy | | 0.4±0.02 | | 0.07±0.07 | | 0.2±0.06 | | 0.2±0.01 | | < LOQ | | 0.0021±0.00003 | | 0.4±0.06 | | 0.2±0.005 | | | 0.4±0.01 | |  | |  |
| - | Er | | 0.2±0.01 | | 0.05±0.05 | | 0.08±0.03 | | 0.09±0.01 | | < LOQ | | 0.0013±0.0001 | | 0.2±0.03 | | 0.1±0.01 | | | 0.2±0.0006 | |  | |  |
| - | Tm | | 0.02±0.001 | | 0.01±0.01 | | 0.01±0.004 | | 0.01±0.001 | | < LOQ | | 0.0001±0.00001 | | 0.02±0.003 | | 0.02±0.001 | | | 0.02±0.0006 | |  | |  |
| - | Yb | | 0.1±0.005 | | 0.04±0.04 | | 0.05±0.02 | | 0.08±0.01 | | < LOQ | | 0.0009±0.0001 | | 0.1±0.02 | | 0.1±0.01 | | | 0.1±0.01 | |  | |  |
| - | Lu | | 0.02±0.0009 | | 0.01±0.01 | | 0.007±0.003 | | 0.01±0.002 | | < LOQ | | 0.0001±0.0000 | | 0.02±0.002 | | 0.02±0.0008 | | | 0.02±0.001 | |  | |  |
| - | Bi | | < LOQ | | < LOQ | | < LOQ | | < LOQ | | < LOQ | | < LOQ | | < LOQ | | < LOQ | | | < LOQ | |  | |  |
| - | | | Th | | 0.05±0.003 | | 0.03±0.004 | | 0.02±0.01 | | 0.02±0.003 | | < LOQ | | 0.0002±0.0001 | | 0.05±0.01 | | 0.007±0.0007 | | 0.04±0.004 | | |  |
| - | | | U | | 0.03±0.002 | | 0.1±0.07 | | 0.01±0.005 | | 0.2±0.02 | | < LOQ | | < LOQ | | 0.04±0.01 | | 0.2±0.01 | | 0.02±0.001 | | |  |
| - | | | Be | | 0.002±0.0001 | | 0.01±0.003 | | 0.0008±0.0001 | | 0.003±0.0003 | | < LOQ | | < LOQ | | 0.003±0.0006 | | 0.005±0.001 | | 0.004±0.0003 | | |  |
| - | | | Ga | | 0.2±0.01 | | 0.04±0.005 | | 0.1±0.04 | | 0.06±0.003 | | < LOQ | | < LOQ | | 0.3±0.04 | | 0.06±0.01 | | 0.3±0.01 | | |  |
| - | | | Y | | 2±0.04 | | 0.8±1 | | 1±0.4 | | 1±0.03 | | 0.004±0.0003 | | 0.03±0.0008 | | 3±0.4 | | 4±0.1 | | 3±0.1 | | |  |
| - | | | Nb | | 0.002±0.00001 | | 0.02±0.005 | | 0.003±0.005 | | 0.008±0.0005 | | < LOQ | | < LOQ | | 0.002±0.0002 | | 0.007±0.002 | | 0.001±0.0002 | | |  |
| - | | | Cs | | 0.003±0.0002 | | 0.001±0.0002 | | 0.001±0.0004 | | 0.002±0.0001 | | 0.0009±0.0001 | | < LOQ | | 0.004±0.0006 | | 0.002±0.0002 | | 0.01±0.001 | | |  |
| - | | | Gd | | 0.6±0.01 | | 0.07±0.07 | | 0.3±0.1 | | 0.2±0.004 | | 0.0008±0.0001 | | 0.003±0.0001 | | 0.8±0.1 | | 0.2±0.004 | | 0.8±0.04 | | |  |
| - | | | Ho | | 0.07±0.001 | | 0.02±0.02 | | 0.03±0.01 | | 0.04±0.0008 | | < LOQ | | < LOQ | | 0.09±0.01 | | 0.05±0.002 | | 0.09±0.004 | | |  |
| - | | | Hf | | 0.005±0.0004 | | 0.006±0.0005 | | < LOQ | | 0.02±0.003 | | < LOQ | | < LOQ | | 0.006±0.001 | | 0.002±0.0005 | | 0.004±0.004 | | |  |
| - | | | Ta | | 0.0003±0.0001 | | 0.0009±0.0001 | | < LOQ | | 0.0005±0.0000 | | < LOQ | | < LOQ | | 0.0003±0.0001 | | 0.0002±0.0000 | | 0.0002±0.0000 | | |  |
| - | | | W | | 0.05±0.06 | | 0.01±0.001 | | 0.003±0.001 | | 0.01±0.0006 | | < LOQ | | < LOQ | | 0.01±0.009 | | 0.007±0.0005 | | 0.006±0.0003 | | |  |
| - | | | Mg | | 1,046±54 | | 946±173 | | 438±228 | | 808±5 | | 104±11 | | < LOQ | | 1,166±83 | | 1,797±186 | | 1,686±344 | | |  |
| - | | | Al | | < LOQ | | 120±20 | | < LOQ | | 60±4 | | < LOQ | | < LOQ | | 279±18 | | 331±99 | | 95±20 | | |  |
| - | | | Mn | | 37±2 | | 8±2 | | 16±8 | | 21±0.4 | | 2±0.2 | | < LOQ | | 36±2 | | 52±5 | | 43±8 | | |  |
| - | | | Sr | | 84±5 | | 54±12 | | 35±18 | | 160±2 | | 48±4 | | 1±0.06 | | 72±5 | | 47±4 | | 110±21 | | |  |
| - | | | La | | 10±10 | | 2±4 | | < LOQ | | 13±22 | | < LOQ | | < LOQ | | 7±5 | | < LOQ | | 202±276 | | |  |

Note: “-”: ICH Q3D not specified; “< LOQ”: below the respective LOQs.
